# Emergency neurosurgical hybrid operating platform for acute intracranial hemorrhage (E-HOPE)

**DOI:** 10.1186/s41016-024-00385-0

**Published:** 2024-12-16

**Authors:** Mingze Wang, Peicong Ge, Yuming Jiao, Xiaofeng Deng, Songlin Yu, Yuha Jiang, Zhi Li, Tao Wang, Hongwei He, Youxiang Li, Xiaolin Chen, Shuo Wang, Yong Cao

**Affiliations:** 1 Department of Neurosurgery, Beijing Tiantan Hospital, Capital Medical University, Beijing, 100070 China; 2 China National Clinical Research Center for Neurological Diseases, Beijing, 100070 China

## Abstract

**Background:**

Precise diagnosis and rapid treatment for acute complex intracranial hemorrhage (ICH) are crucial. The neurosurgical hybrid operating platform integrates traditional open neurosurgery operating room functionalities with endovascular therapy capabilities and is developing in the neurosurgical practice. However, its effect on the emergent complicated neurovascular cases needs pilot exploration.

**Methods:**

In this prospective study, a total of 103 cases of both spontaneous and non-spontaneous ICH were consecutively recruited between June 2019 and June 2023. Demographic data, including age, gender distribution, and types of hemorrhage, were collected. Surgical interventions were tailored based on DSA, including spontaneous and non-spontaneous hemorrhages. Functional outcomes were assessed using the modified Rankin Scale (mRS) preoperatively and postoperatively.

**Results:**

Over the study period from June 2019 to June 2023, a cohort of 103 ICH cases underwent emergency hybrid surgical treatment utilizing the E-HOPE platform. Among these cases, 88 were classified as spontaneous ICH, while 15 were non-spontaneous. The mean age at diagnosis for the entire cohort was 54.0 ± 3.7 years, with a slight predominance of male patients. Spontaneous ICH encompassed a diverse spectrum of etiologies, including arteriovenous malformations, aneurysms, arteriovenous fistulas, cavernous malformations, moyamoya disease, and cryptogenic hemorrhages. Surgical interventions were tailored to address the specific pathology. Notably, improvements in mRS scores were observed in a majority of cases, with some patients experiencing stabilization or deterioration postoperatively. Non-spontaneous cases (*n* = 15) were primarily iatrogenic (*n* = 13) due to tumors adjacent to the internal carotid artery, necessitating stent graft deployment. Surgical approaches, including stent graft deployment and middle meningeal artery embolization, were effective in managing these cases. Postoperative functional outcomes varied depending on the nature of the hemorrhage, with a subset of patients demonstrating improvement in mRS scores while others showed no significant change.

**Conclusions:**

Emergency hybrid surgical treatment utilizing the E-HOPE platform offers promising outcomes for ICH patients. Tailored surgical approaches result in favorable postoperative functional outcomes, highlighting the importance of a multidisciplinary approach in managing these complex cases.

## Background

The hybrid operating room represents a groundbreaking advancement in the field of neurosurgery, merging the functionalities of a traditional operating room with those of an interventional treatment room [1–3]. This integration addresses the inefficiencies inherent in separate approaches to traditional microsurgery and endovascular treatment for complex cerebrovascular diseases [4]. A hybrid operating room could significantly increase the operation success rate and reduce operative duration, mortality rates, and complication rates [5]. By eliminating the need for multiple patient transfers between rooms, the hybrid operating room significantly mitigates the risks posed by repeated anesthesia and transfers [6, 7].

One of the primary benefits of hybrid operating rooms lies in their enhanced intraoperative diagnostic capabilities [8–11]. Equipped with advanced imaging technologies like digital subtraction angiography (DSA) and high-resolution three-dimensional rotational angiography (3DRA), these facilities empower neurosurgeons to precisely pinpoint the sources of bleeding and validate the locations of aneurysms or arteriovenous malformations, etc. [1, 12, 13]. This capability is instrumental in avoiding blind emergency hematoma removal, thus averting potential intraoperative complications.

Intracranial hemorrhage (ICH) emerges as a particularly grave subtype of stroke, characterized by high fatality rates and limited functional recovery [14, 15]. Despite patients initially presenting with mild impairments and intact cognition, the clinical landscape can rapidly evolve due to factors such as hematoma expansion, cerebral edema, and hydrocephalus [14, 16]. The establishment of emergency hybrid surgical platforms offers a promising solution, enabling rapid identification of the underlying cause and targeted, non-blind removal of hematoma, thereby facilitating expedited functional recovery.

Despite the significant potential of hybrid operating rooms, research on their application in emergency neurosurgical interventions remains limited. To address this gap, our study focuses on a series of ICH cases treated using the emergency neurosurgical hybrid operating platform for ICH (E-HOPE) at our center. Specifically, we aim to evaluate its effectiveness in improving surgical outcomes and reducing complications in emergency settings. Through sharing our findings, we aspire to advance the clinical implementation of hybrid emergency surgeries for ICH.

## Methods

The E-HOPE project was initiated at the Beijing Tiantan Hospital, Capital Medical University in 2019 (ClinicalTrials.gov NCT03774017). The Ethics Committee of Beijing Tiantan Hospital has reviewed and approved this study (KY2017-012–02). The project is being carried out in full compliance with all applicable national and international guidelines, including adherence to the Declaration of Helsinki.

### Study design and population

**Fig. 1:**
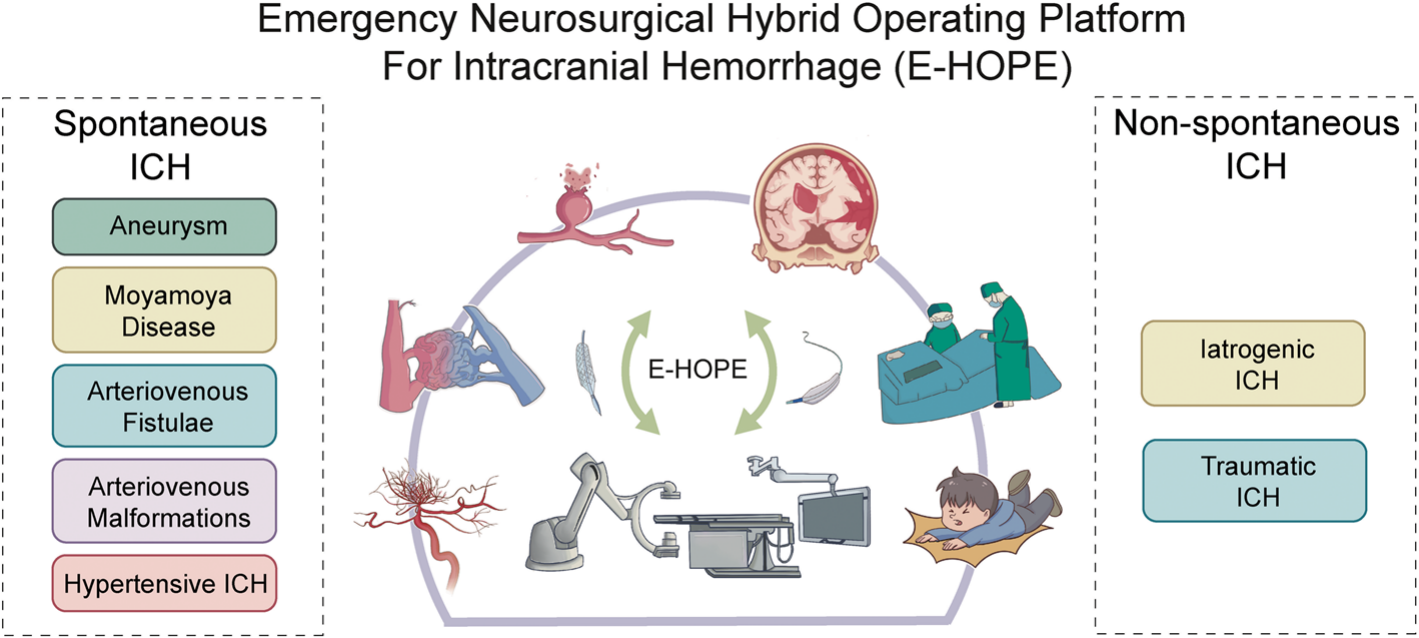
Summary of the major components of the E-HOPE platform

From June 2019 to June 2023, a total of 103 cases of ICH patients underwent emergency hybrid surgical treatment on our E-HOPE platform (Fig. 1). ICH encompasses two primary categories [17]: spontaneous ICH and non-spontaneous ICH. Inclusion criteria: 1. Patients diagnosed with ICH confirmed by CT/CTA imaging. 2 Emergency admission with potential for hybrid surgical intervention, as determined by multidisciplinary evaluation. Exclusion criteria: 1. Patients with severe systemic comorbidities contraindicating surgical intervention. 2. Refusal or inability to provide informed consent.

### Patient management

#### Spontaneous ICH

For patients presenting with spontaneous ICH, initial CT/CTA imaging was performed. Positive CTA findings (e.g., arteriovenous malformations, moyamoya disease, arteriovenous fistulae, or aneurysms) prompted immediate referral to the E-HOPE platform for tailored interventions: arteriovenous malformations: hematoma evacuation, embolization, lesion resection, and angiographic reassessment [4, 12, 18]. Moyamoya disease: hematoma evacuation with intracranial-extracranial revascularization and preservation of superficial temporal arteries. Arteriovenous fistulae: combined hematoma evacuation and fistula embolization or resection, followed by angiographic evaluation. Aneurysms: integrated hematoma evacuation with aneurysm clipping or embolization, followed by angiographic surveillance. In cases with negative CTA findings: hypertensive history with basal ganglia hemorrhage prompted management according to hypertensive ICH protocols. Atypical hemorrhage patterns without hypertensive history were further evaluated using DSA, with subsequent interventions based on findings.

#### Non-spontaneous ICH

Non-spontaneous ICH, predominantly iatrogenic cases during tumor resections, required rapid hemorrhage control. Hybrid interventions included balloon occlusion or covered stent placement on the E-HOPE platform for major vascular injuries.

### Follow-up and outcome evaluation

Following interventions on the E-HOPE platform, patients undergo regular follow-up assessments to evaluate treatment efficacy and monitor for any complications. Imaging modalities, including CT and CTA, are employed during follow-up to assess hematoma resolution, identify any recurrence, and evaluate the efficacy of interventions such as embolization or resection. The primary outcome measure is the improvement in modified Rankin Scale (MRS) scores post-treatment compared to baseline. MRS scores are assessed at predetermined intervals to track functional recovery and assess the impact of interventions on patients’ daily activities. Data were analyzed using SPSS (version 26.0; IBM Corp., Armonk, NY, USA). Continuous variables were expressed as mean ± standard deviation (SD) or median (interquartile range, IQR), and categorical variables as frequencies and percentages. Differences between groups were assessed using the chi-square test for categorical variables. A *p* value < 0.05 was considered statistically significant.

## Results

### Demographic data

In this prospective study, both spontaneous and non-spontaneous ICH were consecutively recruited in E-HOPE platform. Among them, there were 88 cases of spontaneous ICH and 15 cases of non-spontaneous ICH. The mean age at diagnosis was 54.0 ± 3.7 years (range, 50–67 years). The ratio of female to male patients was 1:1.1.

### Spontaneous ICH

Among the 88 cases of spontaneous ICH, the mean age at diagnosis was 38.5 ± 19.0 years (range, 8–77 years). The female-to-male ratio was 1.00:1.84. Among these, 28 cases were negative for DSA and all underwent histopathological examination. Of these, 22 cases were classified as cryptogenic spontaneous ICH and underwent hematoma evacuation surgery; there were 20 patients with a mRS score 5 and 2 patients with a mRS score 4 before the surgery. And 14 patients showed improvement in mRS score, 6 patients showed no improvement, and 2 patients experienced worsening of mRS score, leading to death after the surgery. Four cases were associated with cavernous malformations, undergoing hematoma evacuation followed by cavernous malformation resection. There were 1 patient with a mRS score 5, two with a mRS score 4, and one with a mRS score 3 before the surgery. After surgery, 3 patients showed improvement in mRS score, while 1 patient showed no improvement.

Among the 62 cases with positive DSA findings, the majority were AVMs (39 cases), undergoing single-stage hematoma evacuation, AVM resection, and postoperative surveillance. Prior to surgical intervention, 30 patients presented with a preoperative mRS score of ≥ 3, while 9 patients exhibited a score of < 3. Following surgery, 27 patients demonstrated an improvement in their mRS score, while 9 patients exhibited no discernible change. Regrettably, 3 patients experienced a deterioration in their postoperative mRS score. Aneurysms constituted 12 of the cases, managed through aneurysm clipping followed by hematoma evacuation. Preoperatively, 4 patients displayed a mRS score of ≥ 3, with 8 patients scoring < 3. Subsequent to surgical intervention, 6 patients demonstrated an improvement in their mRS score, while 4 patients remained unchanged. Tragically, 2 patients experienced a decline in their postoperative mRS score.

Arteriovenous fistulas (7 cases) underwent a singular surgical approach involving hematoma evacuation, arteriovenous fistula embolization, and/or resection. Prior to surgery, 1 patient presented with a mRS score of ≥ 3, while 6 patients displayed a score of < 3. Postoperatively, 4 patients exhibited an enhancement in their mRS score, while 2 patients showed no alteration. Alarmingly, 1 patient experienced a worsening of their mRS score following surgery. Moyamoya disease was identified in 4 cases and was treated with a combined approach involving single-stage hematoma evacuation followed by superficial temporal artery wrapping. All 4 patients presented with a preoperative mRS score of 5. Postoperatively, 2 patients demonstrated an improvement in their mRS score, while 2 patients showed no change.

Of the 62 patients with positive DSA findings, 42 were under 50 years old. Among them, 31 showed postoperative improvement, 10 showed no change, and 1 experienced deterioration. For the 20 patients over 50 years old, 10 showed improvement, 8 showed no change, and 2 experienced deterioration. Patients under 50 years of age demonstrated significantly better postoperative outcomes compared to those over 50 years old (*p* < 0.05).

### Illustrative cases

#### Case 1

**Fig. 2:**
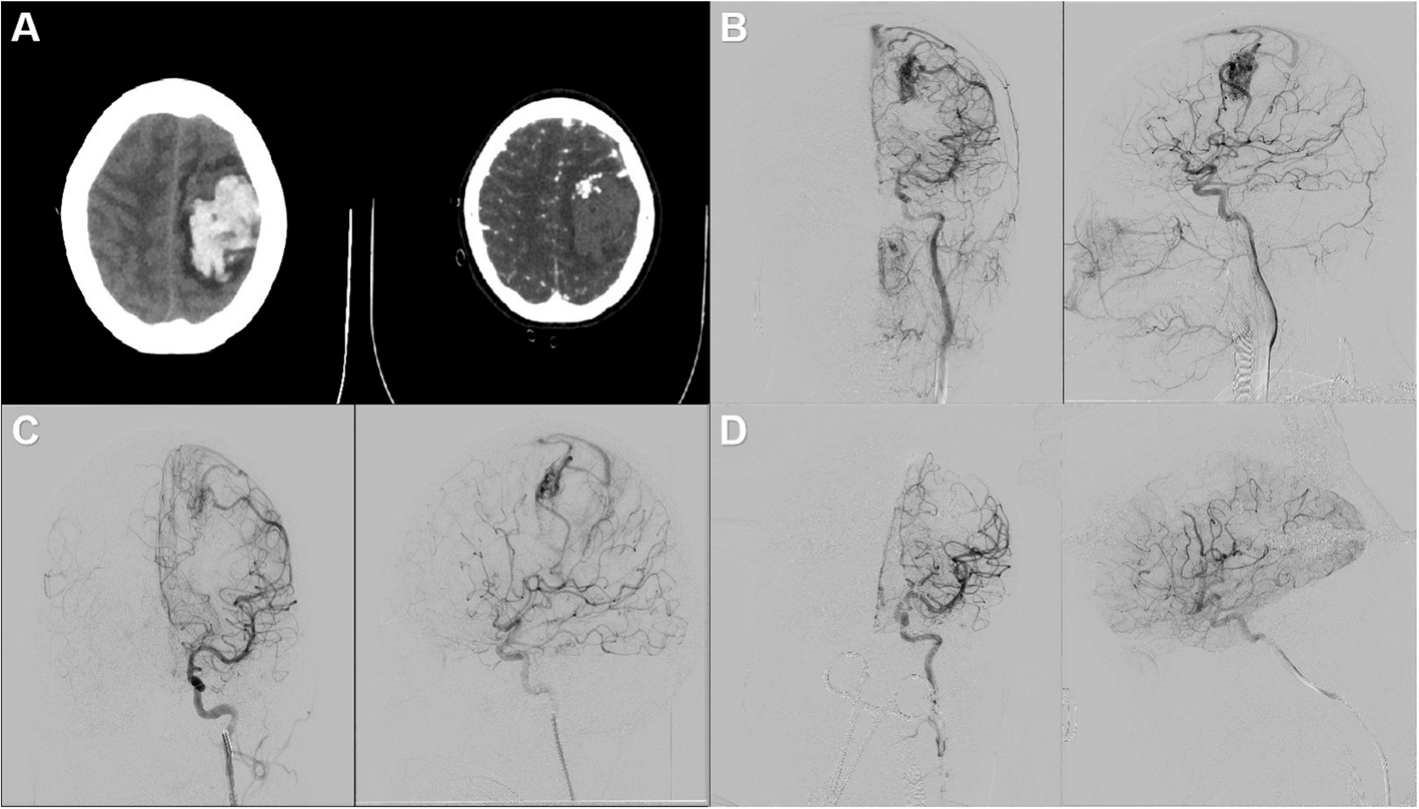
**A** Emergency CT scan indicates hemorrhagic lesions in the left frontal and parietal regions. Suspected left frontal vascular malformation with hemorrhage based on CTA original images. **B** DSA reveals a left frontal vascular malformation supplied by dual branches of the left middle cerebral artery and a single branch of the left anterior cerebral artery, with superficial venous drainage. **C** Embolization of branches supplying the AVM from the middle cerebral artery is performed. **D** Surgical resection and evacuation of the intracranial hematoma are performed. Intraoperative DSA confirms complete obliteration of the lesion post-resection

A 27-year-old female developed severe headache without obvious cause 10 h before admission, which persisted and was not relieved. Meanwhile, there was a decrease in muscle strength of the right upper limb, accompanied by nausea and vomiting of gastric contents. Subsequently, the patient was transported to our hospital by ambulance. Emergency CT scan revealed there are hemorrhagic lesions in the left frontal and parietal regions, as well as subdural hemorrhage in the left frontal temporal region (Fig. 2). The lateral ventricle is compressed, and midline structures are slightly displaced to the right. Emergency CTA results are pending. Considering the original images, left frontal vascular malformation with hemorrhage is suspected. The patient prompts immediate referral to our E-HOPE platform for tailored therapeutic interventions. DSA was performed and revealed a left frontal vascular malformation, supplied by dual branches of the left middle cerebral artery and a single branch of the left anterior cerebral artery, with superficial venous drainage. Following angiography, considering the deeper supply from branches of the middle cerebral artery, arterial embolization of the middle cerebral artery was performed. Then, a surgical resection and evacuation of the intracranial hematoma was performed with neuronavigation and electric cortical stimulation to avoid damaging the superficial eloquence. The lesion was completely resected with the anterior occluded nidus retained. An intraoperative DSA after resection proved the complete obliteration of the lesion.

#### Non-spontaneous intracerebral hemorrhage

Among the 15 cases of non-spontaneous ICH, 13 cases were iatrogenic, with a mean age at diagnosis of 54.0 ± 3.7 years (range, 50–67 years). The female-to-male ratio was 1:1.1. All cases were linked to tumors adjacent to the internal carotid artery, resulting in intraoperative internal carotid artery hemorrhage, necessitating immediate deployment of stent grafts on our E-HOPE platform. Prior to surgery, 9 patients had a modified Rankin Scale (mRS) score ≤ 3, while 3 had a score > 3. Following surgery, 3 patients demonstrated improvement in their mRS score, whereas 9 patients showed no improvement.

Two cases involved chronic subdural hematomas where the bleeding source could not be localized, requiring middle meningeal artery embolization. Before surgery, 1 patient had a mRS score of 5, and 1 had a score of 3. After surgery, 1 patient exhibited improvement in mRS score, while 1 patient showed no improvement.

#### Case 2

**Fig. 3:**
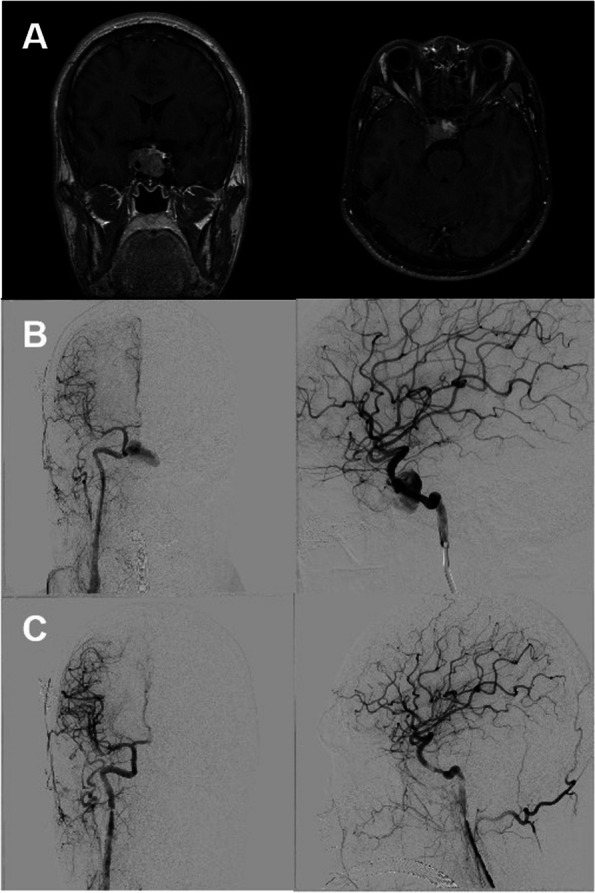
**A** MRI reveals a mass lesion in the sellar region. **B** Intraoperative complication of internal carotid artery rupture during transsphenoidal microscopic surgery for resection of the sellar tumor. **C** Placement of a covering stent on our platform

A 32-year-old male, who 3 months ago noticed changes in facial appearance, thickening of the lips, and enlargement of the extremities, accompanied by hair loss. MRI revealed a mass lesion in the sellar region (Fig. 3). Subsequently, the patient underwent transsphenoidal microscopic surgery for tumor resection in the sellar region. During the surgery, there was a sudden rupture of the internal carotid artery. Emergency intervention was performed using the E-HOPE platform, and a covered stent was inserted into the internal carotid artery for hemostasis.

## Discussion

ICH poses a significant challenge in neurosurgery, marked by considerable morbidity and mortality rates [14, 15, 19]. Managing complex cases often requires a multifaceted approach, combining advanced surgical techniques with precise diagnostic capabilities [17, 20]. In this study, we explored the advantages that the E-HOPE platform brings to ICH across various etiologies, whether spontaneous or non-spontaneous. We observed a reduction in disability and mortality rates among patients. We propose that the E-HOPE platform holds the potential to become a beacon of hope for managing ICH.

### Advantages of E-HOPE platform

#### Comprehensive diagnostic approach

E-HOPE platform enables a comprehensive diagnostic strategy for intricate ICH cases. By leveraging sophisticated imaging modalities such as intraoperative DSA, coupled with meticulous clinical evaluations including neurological assessments and detailed medical histories [7], intraoperative angiography is superior for the detection of remaining remnant vessel abnormalities compared to the alternatives: visual inspection, Doppler ultrasonography, and ICGV [21, 22], we can accurately discern the underlying pathology. This comprehensive approach allows us to identify the etiology of the hemorrhage, assess the extent of the damage, and determine the most appropriate course of action for each individual patient, whether it involves surgical intervention, medical management, or a combination of both. A pivotal advantage of the E-HOPE platform in ICH treatment lies in its seamless integration of endovascular and open surgical procedures. This integration eliminates the need for time-consuming patient transfers between the operating room and angiography suite [1, 5, 23]. Consequently, previously unfeasible rescue surgeries within standard angiography suites are now facilitated, as supported by numerous research findings [1, 3]. This streamlined approach not only saves time but also optimizes patient outcomes by providing timely and appropriate interventions, essentially equating to saving precious brain time and salvaging critical brain function.

#### Integrated therapeutic solutions on E-HOPE platform

Following the confirmation of diagnosis, our E-HOPE platform offers integrated therapeutic solutions for complex vascular lesions such as AVMs and AVFs. Utilizing state-of-the-art surgical modalities including microsurgical techniques, endovascular procedures, and intraoperative DSA, we conduct single-stage resections of these lesions with precision and efficiency [24–28]. Traditionally, craniotomy procedures involved extravascular hemostasis for vascular lesions, while interventions focused on intravascular hemostasis. However, our E-HOPE platform revolutionizes this approach by enabling both extravascular and intravascular hemostasis simultaneously. This unique capability allows us to comprehensively address vascular lesions, maximizing treatment effectiveness and minimizing surgical invasiveness. By combining various treatment modalities within a single surgical session, we minimize the need for multiple surgeries, reduce overall treatment time, and optimize patient outcomes [27]. Additionally, our multidisciplinary team approach ensures seamless coordination among neurosurgeons, interventional neuroradiologists, neuroanesthesiologists, and critical care specialists, providing comprehensive care throughout the treatment process.

Moreover, the integration of intraoperative DSA enables real-time visualization of vascular structures and blood flow dynamics, allowing for precise localization and characterization of vascular lesions. By overcoming intraoperative challenges, such as hematoma-induced distortion of structures, the platform allows for repeated DSA evaluations during surgery. This enables continuous real-time visualization and the ability to make immediate adjustments to the surgical plan. Such capabilities are particularly valuable in managing complex vascular diseases, where timely intervention is essential to preserve neurological function. This intraoperative imaging guidance not only enhances surgical precision but also improves patient safety, ultimately contributing to better treatment outcomes and a reduction in postoperative complications.

#### Management of uncommon iatrogenic complications

Our experience with the E-HOPE platform extends beyond conventional ICH cases to encompass the management of rare iatrogenic complications, including intraoperative internal carotid artery rupture. Intraprocedural arterial perforation (IPAP) is a potentially dismal complication of iatrogenic therapy with high mortality and morbidity rates [29]. Through prompt recognition and intervention, often utilizing endovascular techniques such as coil embolization or stent-assisted coiling, we effectively address these challenging scenarios, minimizing the risk of further neurological damage and optimizing patient outcomes. Additionally, our institution’s robust quality assurance and patient safety protocols ensure the early detection and management of iatrogenic complications, including close postoperative monitoring in specialized neurocritical care units and prompt initiation of rehabilitative interventions to optimize functional recovery.

### Limitation

While our study underscores the promising outcomes associated with the E-HOPE platform in managing complex ICH cases, certain limitations should be acknowledged. Despite demonstrating advantages such as precise diagnosis and integrated therapeutic modalities, including simultaneous intravascular and extravascular hemostasis, our study’s single-center design and relatively small sample size may limit the generalizability of our findings. Additionally, incomplete data, potential selection biases, and the heterogeneity of cases further complicate the interpretation of outcomes. Moreover, the resource-intensive nature of implementing the E-HOPE platform and integrated therapeutic modalities may pose challenges for broader implementation in diverse healthcare settings. Future prospective studies with larger sample sizes, longer follow-up periods, and standardized protocols are warranted to validate our findings and address these limitations, ultimately elucidating the full potential of the E-HOPE platform in optimizing outcomes for patients with complex ICH.

## Conclusions

In conclusion, combined surgical interventions offer distinct advantages in navigating complex ICH cases, facilitating precise diagnosis and integrated therapeutic modalities. Through the utilization of our E-HOPE platform and a multidisciplinary team approach, we adeptly address a spectrum of vascular lesions and rare iatrogenic complications, reaffirming our dedication to excellence in neurosurgical practice.

## Data Availability

The datasets during and/or analyzed during the current study are available from the corresponding author on reasonable request.
